# Cardiogenic shock due to a spontaneously ruptured pheochromocytoma: a rare but life-threatening event—a case report

**DOI:** 10.1007/s00392-022-02115-x

**Published:** 2022-10-10

**Authors:** Anna-Lena Weber, Tobias Jonathan Pfeffer, Holger Leitolf, Bastian Ringe, Heiner Wedemeyer, Johann Bauersachs, Andreas Schäfer

**Affiliations:** 1 Department of Gastroenterology, Hepatology and Endocrinology, Hannover Medical School, Hannover, Germany; 2 Department of Cardiology and Angiology, Hannover Medical School, Hannover, Germany; 3 Department of General, Visceral and Transplantation Surgery, Hannover Medical School, Hannover, Germany

Sirs,

A 54-year-old patient was admitted to the hospital’s emergency department with ongoing cardiopulmonary resuscitation. Before admission, the patient presented to another hospital several hours before due to acute nausea, vomiting and severe back pain. On admission to the external hospital, a leukocytosis of 25 × 1000/µl (reference range < 10 × 1000/µl), significantly increased d-dimers of 8.26 mg/l (reference range < 0.5 mg/l) and an increased troponin T of 2.550 ng/ml (reference range < 0.014 ng/ml) were found. Computed tomography of the chest excluded an aortic dissection and pulmonary artery embolism. A retroperitoneal hematoma originating from the right renal compartment was found incidentally. Due to a significant increase of troponin T to 9.690 ng/ml, a myocardial infarction was suspected and transfer to another hospital with available cardiac catheterization was planned. Before the transfer, a hemodynamically unstable supraventricular tachycardia became apparent, so that Metoprolol (5 mg) was administered. Shortly after drug administration, the patient suffered cardiac arrest caused by pulseless electrical activity (PEA).

Since the patient remained in refractory circulatory arrest, she was transferred to our hospital (distance between hospitals 20 km) with ongoing LUCAS^®^-assisted mechanical resuscitation. Upon arrival, venoarterial extracorporeal membrane oxygenation (VA-ECMO) was immediately implanted to provide extracorporeal cardiopulmonary resuscitation (eCPR). The cumulative time from collapse/ start of resuscitation to VA-ECMO implantation was 60 min. Pre-existing disease conditions were neurofibromatosis type 1 and grade II goiter. The only pre-medication was L-thyroxine 100 µg/day. The family’s general practitioner had carried out regular medical history checkups, which so far had not revealed any further abnormalities. A cardiological checkup had not been performed previously.

**Fig. 1:**
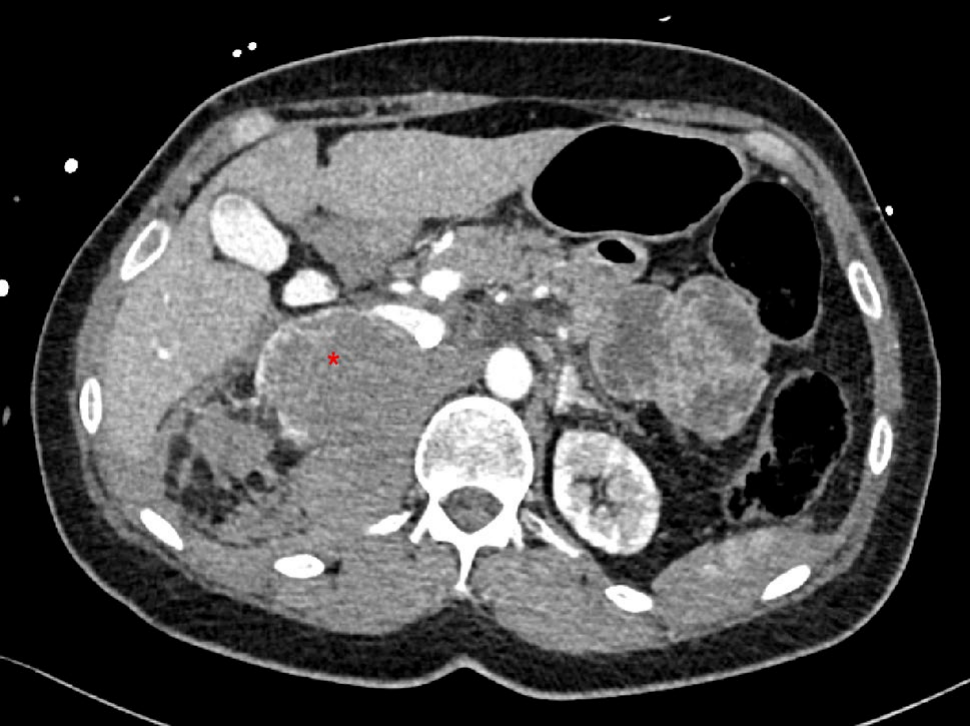
CT scan after providing sufficient circulation with VA-ECMO. *Is marking the 5 × 5 cm mass of the right adrenal gland

Sufficient circulation was immediately provided by VA-ECMO. Transthoracic echocardiography revealed an acontractile heart. A CT scan was performed immediately showing a 5 × 5 cm mass of the right adrenal gland with partial rupture and a retroperitoneal hematoma in the renal compartment (Fig. 1). Subsequent coronary angiography ruled out coronary artery disease. Due to the severely restricted biventricular cardiac function and potential LV overload by retrograde arterial ECMO flow, an Impella CP microaxial pump was implanted to actively unload the LV (ECMELLA concept). Laboratory chemistry detected an increased CK of 1571 U/l (reference range < 145 U/l), an increased CK-MB of 283 U/l (reference range < 24 U/l) and a significantly increased troponin T of 8093.0 ng/l (reference range < 14 ng/l). Renal function was moderately reduced with an eGFR of 41 ml/min/1.73 m^2^. The blood count revealed progressive leukocytosis of 34.1 × 1000/µl (reference range < 10.2 × 1000/µl) and a hemoglobin of 11.1 g/dl. The initial arterial blood gas analysis showed severe combined metabolic and respiratory acidosis with a pH of 6.92 and a lactate of > 15 mmol/l during resuscitation. On intensive care unit, the patient had hypertensive blood pressure values, despite fulminant cardiogenic shock requiring mechanical circulatory support, prompting initiation of antihypertensive therapy with urapidil.

Due to the known neurofibromatosis type 1, the unclear partially ruptured right adrenal mass and the cardiogenic shock with still unclear cause, a catecholamine-induced cardiomyopathy associated with pheochromocytoma was suspected. Meta- and normetanephrine as well as adrenaline and noradrenaline in the serum were determined, even if the value was limited under these circumstances. The adrenaline level was significantly increased at 1003 ng/l (reference value < 84 ng/l) and the noradrenaline level at 1388 ng/l (reference value < 420 ng/l). The metanephrines were also significantly increased at 1068 ng/l (reference value < 90 ng/l) and normetanephrine at 1071 ng/l (reference value < 200 ng/l).

Based on these findings, it was decided to surgically remove the adrenal mass while the patient was still on ECMELLA support. Subsequent histopathological examination of the surgical specimen confirmed the suspected diagnosis of a ruptured, centrally bleeding, necrotic pheochromocytoma. With the pheochromocytoma’s removal, the antihypertensive therapy had to be ended and, due to profound hypotension, vasopressor therapy had to be started. Cardiac function recovered, so that both VA-ECMO and Impella were successively weaned and explanted. During further course, circulatory support therapy was no longer necessary, but there were recurrent hypertensive crises. After a dilation tracheostomy was performed for further weaning from mandatory ventilation, sedation could be gradually tapered off. Fortunately, the patient was awake afterwards and reacted adequately. There was pronounced generalized muscle weakness in the sense of a critical illness polyneuropathy, so the patient was transferred to an appropriate specialist clinic for further neurological-intensive care rehabilitation.

A pheochromocytoma is a rare, catecholamine-producing tumor that arises predominantly from the chromaffin cells of the adrenal medulla. Approximately, 0.2–0.6% of hypertensive patients are affected [1]. In rare cases, pheochromocytoma is part of a hereditary syndrome, such as neurofibromatosis type 1 (NF1). Up to 6% of patients with NF1 develop pheochromocytoma, whereby most of them are asymptomatic [2, 3]. The symptoms are characterized by an overproduction of catecholamines and primarily include headaches, palpitations and sweating [1]. A feared complication is the pheochromocytoma crisis, which manifests itself as a hypertensive crisis, but in the worst case can also be accompanied by circulatory shock and multi-organ failure [4]. The rupture of a pheochromocytoma is a very rare event with a high mortality rate. So far, there are only a few cases described worldwide [5], and to our knowledge, there is no described case with a refractory cardiac arrest cause of a ruptured pheochromocytoma with need of eCPR and following biventricular assist devices. The rupture could be caused by rapid tumor growth or bleeding into the tumor. The rupture leads to a massive release of catecholamines, which, as described in our case, led to catecholamine-induced cardiomyopathy and cardiogenic shock with consecutive cardiac arrest. Certainly, the administration of the beta blocker metoprolol had an aggravating effect, due to loss of beta-mediated vasodilatation. Therefore, beta blocker should only use in combination with alpha blocker in pheochromocytoma.

If a pheochromocytoma is suspected, the meta- and normetanephrines in serum and in 24-h urine samples should be determined. It should be noted, however, that endogenous catecholamine production occurs, especially in critically ill patients who require mechanical circulatory support, regardless of the presence of a pheochromocytoma [6]. Contrast-enhanced computed tomography, which is usually readily available, is sufficient to localize the tumor. In this case, the combination of measured catecholamines in serum and the imaging findings was sufficient to establish the diagnosis. Further diagnostic tests were not necessary. Due to the high number of asymptomatic pheochromocytomas, patients with NF1 should be screened from the age of 40 [3].

Therapy depends on the severity of the disease and in our case initially consisted of supportive circulatory therapy for refractory cardiogenic shock. The early use of combined mechanical circulatory support from VA-ECMO and an Impella microaxial pump is an option for such patients [7]. However, in pheochromocytoma crisis, there are only a few cases that reported the use the ECMELLA concept [8–10]. Surgical resection of the pheochromocytoma should be envisaged as causal therapy. The optimal time is controversial. On the one side, early resection is recommended despite the lack of alpha-blockade, on the other, stabilization of the patient initially seems sensible [4].

In our case, the patient had to be stabilized first due to refractory cardiogenic shock. Subsequently, an operative resection of the tumor was successful under the protection of combined biventricular mechanical circulatory support.

This case report shows a very rare cause of cardiogenic shock. Cardiac causes such as myocardial infarction or myocarditis are often found in patients with cardiogenic shock. In the case of ambiguous findings in particular, however, extracardiac diseases should be considered and extended diagnostics should be carried out.
